# Immunohistological characterization of equine synovial tissue in metacarpophalangeal joints of different ages and osteoarthritis status

**DOI:** 10.1016/j.ocarto.2026.100819

**Published:** 2026-05-14

**Authors:** Giulia Liso, Karsten Winter, Theresa Jenny Triebe, Reiner Ulrich, Walter Brehm, Bettina Wagner, Christiane Liliane Schnabel, Antonia Troillet, Susanne Pauline Roth

**Affiliations:** a Saxonian Incubator for Clinical Translation (SIKT), Leipzig University, Leipzig, Germany; b Veterinary Teaching Hospital, Department for Horses, Faculty of Veterinary Medicine, Leipzig University, Leipzig, Germany; c Institute of Anatomy, Leipzig University, Leipzig, Germany; d Institute of Veterinary Pathology, Faculty of Veterinary Medicine, Leipzig University, Leipzig, Germany; e Department of Population Medicine and Diagnostic Sciences, College of Veterinary Medicine, Cornell University, Ithaca, NY, United States of America; ∥ Institute of Immunology, Faculty of Veterinary Medicine, Leipzig University, Leipzig, Germany

**Keywords:** Horse, Foal, Degenerative joint disease, Synovitis, Macrophages, Immunofluorescence, Multiplex assay

## Abstract

**Objective:**

Synovial macrophages' role in equine osteoarthritis (OA) is not well understood, despite horses being a model for human degenerative joint disorders. Equine synovial macrophages’ characterization is limited and age-related changes are not described. This study aimed to evaluate surface marker distribution and expression from potential synovial tissue macrophages in foals, adult non-affected, and OA-affected horses.

**Methods:**

Surface markers (CD14, CD16, CD206) were quantitatively evaluated on dorsal villous synovial biopsies from the metacarpophalangeal joint of foals (F), non-affected (control; CTRL), and OA-affected adults (OA) using immunofluorescence histology and computed image analysis. Samples were analyzed in three regions: intimal cell layer (ICL), subintimal tissue layer of the villous region (SI–V), and subintimal tissue layer not associated with the villous region (SI–N). Synovial fluid protein, cytokine, and chemokine concentrations, and hematoxylin-eosin (HE) staining were performed to assess joint disease state.

**Results:**

Marker-associated areas were increased in ICL and SI–V compared to SI–N for all three groups. The CD14^+^ area was increased in the CTRL when compared to the OA and F groups (ICL, SI–V). CD16^+^ areas were similar in all groups, while CD206+ area was larger in foals compared to CTRL and OA groups (ICL, SI–V). Synovial fluid cytokine and chemokine concentrations did not differ between groups.

**Conclusions:**

Computed analysis of immunofluorescence staining of CD14, CD16, and CD206 on equine synovial tissue revealed region specific marker expressions. While an altered marker expression in relation to age and degenerative joint disease was shown, further investigation is required to determine marker significance for identifying equine synovial tissue macrophages.

## 1 Introduction

As with humans, a key pathogenetic process in equine osteoarthritis (OA) is represented by low-grade synovitis, driven by synovial macrophages [[1], [2], [3]]. Although human synovial macrophages have been extensively studied in healthy and diseased joints, their detailed functions, developmental origin and the microenvironmental factors determining their functional status are still incompletely understood [[4], [5], [6], [7]]. Despite considerable research in recent years, the M1/M2 paradigm has proven insufficient for clear cell characterization to include cell function and plasticity, and for the reliable distinction of synovial macrophage subsets or states [8]. In particular, the diverse and inconsistent use of markers for the M1/M2 subtypes has been shown to be insufficient to identify macrophage subsets consistently.

Human studies histologically evaluating synovial tissue macrophages have been performed on OA patients undergoing total knee replacement surgery and included surface markers CD68 (total macrophage population), CD80 and CD86 (pro-inflammatory), and CD206 (anti-inflammatory) [[9], [10], [11]]. Although moderate-grade synovitis cases are characterized by an overall high CD68 percentage [9], expression and tissue localization of the remaining markers revealed large interindividual variations [9,10] and were not sufficient to clearly distinguish OA and rheumatoid arthritis [11].

In horses, studies to characterize synovial macrophages and their pathogenetic role in OA have been severely limited because identification and precise characterization of definitive markers for equine synovial macrophages remain challenging and controversial [[12], [13], [14], [15], [16]]. In various equine tissues, macrophages have been found to express the lipopolysaccharide co-receptor CD14, making it a potentially useful macrophage marker in horses. OA-affected equine joints exhibit elevated numbers of CD14^+^ macrophage-like cells compared to non-affected joints, indicating a potential link between CD14 expression and joint pathology [14]. CD16, known as FcγIII receptor, is observed on equine non-classical blood monocytes (CD14loCD16+) [[17], [18], [19]] and on most equine bronchoalveolar lavage macrophages [13], but has not yet been described on equine synovial tissue cells. Available literature suggested that horses (like many other non-primates) express only a transmembrane form of CD16 and no functional equivalent of the human CD16A and CD16B isoforms has been conclusively identified [17]. Synovial macrophage-like cells widely express CD206 [1,20]. The regulation of CD206 was demonstrated in a report on equine asthma [21] but not in naturally occurring OA [14]. CD206, a mannose receptor involved in scavenging responses, is expressed by equine blood monocytes [22]. To date, a single study has investigated the potential pro- and anti-inflammatory dynamics of equine synovial macrophages in naturally occurring osteoarthritis, based on CD86, CD206, and CD14 expression [14].

Therefore, we aimed to further evaluate surface markers of potential equine synovial macrophages, including CD16. The current study included foals and adult horses, with and without naturally occurring OA, and evaluated potential synovial macrophage phenotypes in relation to joint maturation and degeneration. To further investigate the synovial microenvironment, synovial fluid cytokines and chemokines, as well as total protein concentrations were analyzed to test the following hypotheses: (1) The synovial status of foals and the microenvironment of OA in adult horses alter the expression of CD14, CD16, and CD206 on potential synovial macrophages as well as their intimal cell density, and (2) concentrations of pro- and anti-inflammatory synovial fluid cytokines and chemokines correspond to macro- and microscopic joint assessment.

## 2 Method

### 2.1 Synovial tissue and synovial fluid sampling

The study was conducted using synovial fluid and synovial tissue samples from the metacarpophalangeal joints of foals (F) (n = 10) and adult horses euthanized for reasons unrelated to the study (n = 20). Samples from adult horses were allocated based on macroscopic assessable joint degeneration (OARSI recommendations for equine OA assessment [23]) to non-affected adults (control; CTRL) (n = 11) and OA-affected adults (OA) (n = 9). All procedures were approved by the local ethics committee (EK 20/2023).

Synovial fluid aspiration (2 ml) was performed aseptically using a 20G-hypodermic needle from the lateral recess of the joint, aliquoted (2 ml sample tube containing tripotassium ethylenediamine tetra-acetic acid [K3EDTA]; SARSTEDT AG & Co. KG), and subsequently centrifuged at 3000×*g* for 10 min. Total protein (TP) was analyzed with a refractometer (Vet Device, Clinical Refractometer, S.P. & U.G., ATC) and the synovial fluid supernatant was stored at – 80 °C for subsequent cytokine and chemokine analysis (Animal Health and Diagnostic Center at Cornell University College of Veterinary Medicine, USA).

Thereafter, the joint capsule was dissected as previously described [24] and the articular cartilage was evaluated macroscopically for signs of OA according to the OARSI recommendations for equine OA assessment [23]. Joints were classified as OA-affected (OA) when a mean total macroscopic score of 3 or higher was observed representing the articular cartilage parameters wear lines, erosions, and palmar arthrosis. Two 8 mm biopsy punch samples were obtained from the dorsomedial and dorsolateral villous part of the synovial membrane of all joint capsules. These samples were randomly assigned for further processing: sample A was allocated for immunofluorescence staining, and sample B was allocated for hematoxylin-eosin (HE) staining. Samples A were immediately transferred into Hank's balanced salt solution (HBSS, Gibco, Life Technologies Limited) and processed for storage at - 80 °C according to standard protocols [25,26]. Samples B were fixated in 4 % formaldehyde (ROTI® Histofix 4 %, phosphate-buffered, pH 7, Carl Roth GmbH + Co. KG) for 24–72 h.

### 2.2 Histological processing

For direct immunofluorescence staining, frozen samples (A) were sectioned at 5 μm thickness. Slides were stained using antibodies targeting the following cell surface markers: CD14 (anti-equine CD14, clone 105, mouse, monoclonal IgG1; Wagner Laboratory, Cornell University) [12], CD16 (anti-equine CD16, clone 59G5, mouse, monoclonal IgG1; Wagner Laboratory, Cornell University) [17], and CD206 (anti-human CD206, clone 3.29B1.1, mouse, monoclonal IgG1; Beckman Coulter GmbH). Immunofluorescence staining procedures included the double staining of CD14 and CD16 as well as of CD14 and CD206. The antibody panel and processing are specified in Table 1. Immunofluorescence staining of CD16 and CD206 was pre-evaluated on equine blood monocyte-derived macrophages (Supplementary Figure 1). Anti-CD206 was conjugated with phycoerythrin (PE) by the manufacturer, the primary antibodies detecting CD14 and CD16 were conjugated in house with fluorochromes using sulfo–NHS–ester conjugation kits: CD14 with AlexaFluor 647 and CD16 with AlexaFluor 555 (Thermo Fisher Scientific, Life Technologies). Cell identification was performed with DAPI nuclear counterstain (Thermo Fisher Scientific, Life Technologies). Negative controls included isotype controls to evaluate potential non-specific binding of the respective primary antibody. Immunofluorescence-labeled samples (A) were stored at 6–8 °C in the dark, and the fluorescence signal was analyzed within 2 h after finishing the immunofluorescence staining process. Samples for standard histology (B) were sectioned at 5 μm thickness and HE-staining was performed.

**Table 1:** Detailed antibody panel used for immunofluorescence staining of synovial tissue samples (A).

Marker	Antibody	Fluorochrome	Company	Isotype control	Exposure time
CD14	Anti-equine CD14, clone 105^a^	Alexa 647	Wagner Laboratory, Cornell University	Alexa647 mouse IgG1	600 ms
CD16	Anti-equine CD16, clone 59G5^a^	Alexa 555	Wagner Laboratory, Cornell University	Dye 550 mouse IgG1	200 ms
CD206	Anti-human CD206-PE, clone 3.29B1.1^a^	PE	Beckman Coulter GmbH	PE, mouse IgG1	200 ms

a All primary antibodies are monoclonal mouse IgG1.

### 2.3 Histological analysis and semi-automated image analysis

All stained synovial tissue sections (A, B) were digitized using a Pannoramic Scan II (3D HISTECH Ltd.). Images were exported from the slide scanner data sets using CaseViewer (version 2.3, 3D HISTECH Ltd.) as gray-scale fluorescence channels for samples A, and RGB brightfield images for samples B, respectively.

HE-stained sections (B) were classified according to the histological grading system previously published for equine synovial tissue to assign a histological score corresponding to the macroscopic score obtained during dissection [23]. This classification was performed in a blinded manner by a board-certified member of the European College of Veterinary Pathologists (RU). To additionally define subintimal vascularization, the circumference of subintimal vessels was determined for HE-stained samples by labeling separate layers using Photoshop (version 22.1.1, Adobe).

**Fig. 1:**
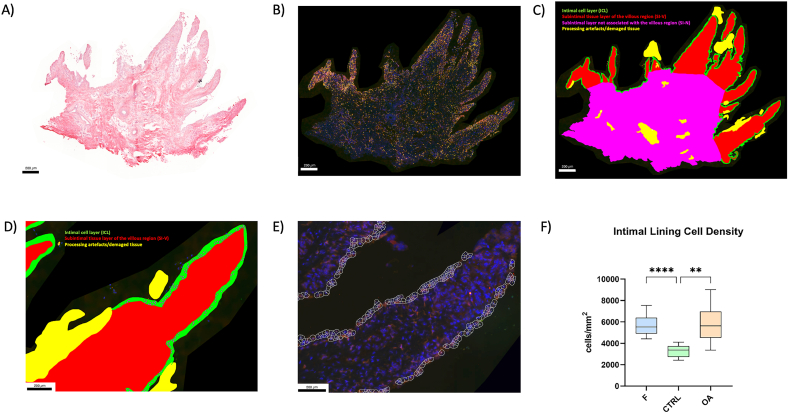
**(A)-F))**. Serial synovial tissue sections of the metacarpophalangeal joint of a foal including HE-stained section **A)**, immunofluorescence-stained section **B)**, digitized section with manually marked regions of interest **C)**, and with manually punctuated lining cells **D)**. Three tissue regions were delineated **C)**: the ICL colored in green, the SI-V colored in red, and the SI-N colored in pink. Tissue processing artefacts or damaged tissue that was inappropriate for analysis was colored yellow. The lining cells were punctuated in magenta to quantify the intimal lining cell density **D)**. Automated analysis of intimal lining cell density **E)**, and boxplots of the intimal lining cell density given in number of cells/mm^2^**F)**. Based on DAPI nuclear stain (blue) individual cells of the ICL were punctuated, and individual cell masks were computed **E)**. The intimal lining cell density was analyzed in the F group (foals), the CTRL group (control; non-affected adults), and the OA group (OA-affected adults). Compared to the CTRL group, there were significantly higher numbers of intimal lining cells per mm^2^ in the F and OA groups. (∗∗ *p* < 0.010; ∗∗∗∗*p* < 0.0001).

Analysis of immunofluorescence-labelled samples (A) was conducted by manually delineating three tissue regions of interest: the intimal cell layer (ICL), the subintimal tissue layer of the villous region (SI–V), and the subintimal layer not associated with the villous region (SI–N) (Fig. 1A–C). To evaluate the ICL and SI–V, the synovial villus was identified based on histological morphology, characterized by a greater height than width [24]. Within each synovial villus, the ICL was defined as the apical two to three cell-thick layers. The density of intimal lining cells (total synovial cells) was assessed by manually marking all individual cells present in the ICL, as indicated by DAPI nuclear staining. The portion of the synovial tissue section not associated with the villous region was designated as SI–N (Fig. 1A–C). All subsequent automated image processing steps were carried out using Mathematica (version 11.3, Wolfram Research Inc., Champaign, IL). Locally adaptive thresholding (LocalAdaptiveBinarize, filter width 50 pixels) was applied to distinguish signals of positive cells from the image background, resulting in binary masks. Cells with co-localized signals (CD14^+^CD16^+^; CD14^+^CD206+) were identified by multiplication of the respective binary masks. Subsequently, for each cell mask the relative amount of signal areas (%/mm^2^) was computed. Center positions of fluorescence-labelled intimal lining cells were extracted to assign individual cells to the respective tissue region of interest. Cell areas and respective individual masks were computed based on distance transformation and watershed segmentation, with an empirically determined average cell radius of 15 pixels (Fig. 1D–E).

### 2.4 Cytokine and chemokine analysis

Cytokine (TNF-α, IL-1β, IL-17A, IFN-α, IFN-γ, IL-4, IL-10) and chemokine (CCL2, CCL3, CCL5, CCL11) concentrations from synovial fluid samples were evaluated by fluorescent bead-based multiplex assays using validated equine monoclonal antibodies as previously described (Animal Health Diagnostic Center, Cornell University) [[27], [28], [29], [30], [31]]. TNF-α, IL-1β, IL-17A, IFN-α, and IFN-γ are classified as primarily pro-inflammatory cytokines. In contrast, IL-4 and IL-10 are also involved in anti-inflammatory processes. The four CCL chemokines exert immunomodulatory effects and/or chemotactic effects by immune cells, such as monocytes, macrophages, and/or lymphocytes [28].

### 2.5 Statistical analysis

Statistical data analysis was performed using Prism version 9.1.2.226 (GraphPad Software by Dotmatics). Descriptive statistics were calculated, and boxplots were generated. The data were subjected to a normality test (Shapiro-Wilk-Test). The mean value with standard deviation (mean ± SD) was specified for normally distributed data, and the median with the 25 %–75 % quantiles was specified for non-normally distributed data. Normally distributed data were analyzed using either a *t*-test or an ANOVA with a post-hoc Tukey test to adjust the *p*-values for multiple comparisons. Non-normally distributed data were examined using the Kruskal-Wallis test with Dunn's post-hoc test to adjust the *p*-values for multiple comparisons. Statistical significance was set at *p* < 0.050.

## 3 Results

### 3.1 Histological evaluation of synovitis signs and vascularization (samples B)

**Fig. 2:**
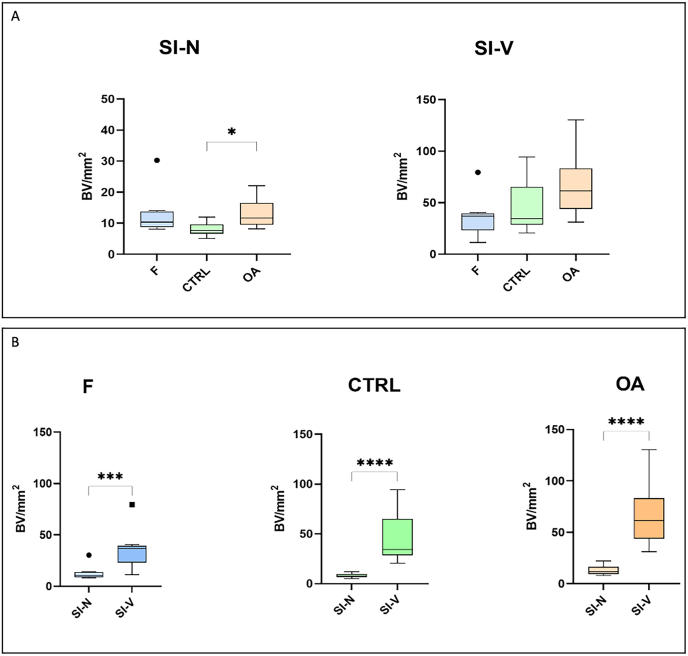
Histological analysis of vascularization. Boxplots demonstrating number of blood vessels (BV) evaluated in the F group (foals), the CTRL group (control; non-affected adults), and the OA group (OA-affected adults) given in number of BV/mm^2^ within specific tissue regions: the SI-N, and the SI-V. Significant differences are indicated (∗*p* < 0.050; ∗∗∗*p* < 0.001; ∗∗∗∗*p* < 0.000).

An overview of included horses and their classification according to the macroscopic OARSI score is given in Table 2. The histological analysis of the synovial tissue samples stained with HE revealed a significant difference for the OARSI parameter of cellular infiltration, which was higher in the OA group compared to the CTRL group (*p* < 0.040) (Table 3). When evaluating vascularization, represented by the number of blood vessels (BV) per mm^2^ in HE-stained tissues, a higher number in the SI–V was noted compared to the SI–N, evident across all three groups (F group: *p* < 0.001; CTRL and OA groups: *p* < 0.001). Although the lowest vascularization was observed in the SI–N when comparing tissue regions, the OA group demonstrated an increase compared to the CTRL group only within the SI–N (*p* < 0.050) (Fig. 2).

**Table 2:** Overview (age, sex) of included foals (F), macroscopically non-affected adult horses (control; CTRL), and adult horses classified as OA-affected (OA). Six of the ten included foals showed signs of septicemia. For the F group, no macroscopic OARSI scoring was performed (not applicable; N/A). The macroscopic OARSI score included the parameters wear lines, erosions, and palmar arthrosis, each with a score grade ranging from 0 (unaffected) to 3 (severely affected) [23]. In the CTRL group, wear lines, erosions, and palmar arthrosis were graded as maximal with grade 1 out of 3 (mild changes). In the OA group, only one sample had a mean total score of 3 (the minimum mean total score required for classification in the OA group); all other samples of the OA group ranged from a mean total macroscopic OARSI score of 4–8^#^. For the parameters wear lines and palmar arthrosis in the OA group, there was only one sample each with a score of 0^§^.

Group	Mean age ± standard deviation [SD]	Sex			Macroscopic OARSI score [23] (score 0 [unaffected] to score 3)			
		Female	Male	Gelding	Wear lines	Erosions	Palmar arthrosis	Mean total score ± standard deviation [SD]
F (*n* = 10)	45 days ± 41 days	6	4	0	N/A	N/A	N/A	N/A
CTRL (*n* = 11)	9 years ± 6 years	7	2	2	≤1 (mild changes)	≤1 (mild changes)	≤1 (mild changes)	1.5 ± 0.5
OA (*n* = 9)	18 years ± 6 years	4	0	5	0–3^§^	1–3	0–3^§^	5.4 ± 2^#^

**Table 3:** Median values with corresponding *p*-values from the histological analysis of synovial tissue samples (B) regarding signs of synovitis (cellular infiltration, vascularity, intimal hyperplasia, subintimal edema, subintimal fibrosis) obtained from macroscopically non-affected adult horses (control; CTRL) and adult horses classified as OA-affected (OA) according to the histological OARSI-score parameters [23].

Group	Cellular infiltration	Vascularity	Intimal hyperplasia	Subintimal edema	Subintimal fibrosis
CTRL	0.5	3	1	0.5	2.5
OA	1	2	1	1	2.5
*p*-value	<0.050	>0.050^a^	>0.050^a^	>0.050^a^	>0.050^a^

a All not significantly different between the groups.

### 3.2 Immunofluorescence analysis of cellularity and surface markers

The assessment of intimal lining cell density using DAPI-based staining in the ICL, revealed elevated values in both the F (median 5532 cells/mm^2^, 5044–6290.3 cells/mm^2^) and OA groups (median 5641.36 cells/mm^2^, 4885.1–6878.7 cells/mm^2^). In contrast, the CTRL group exhibited the lowest cell density, with these differences reaching statistical significance when compared to both the F (*p* < 0.001) and OA group (*p* < 0.005) (Fig. 1F).

Tissue region-dependent CD14 distribution: The relative CD14^+^ areas in the SI–N were lower compared to the SI–V and ICL regions across all three groups. Larger CD14^+^ areas were found in the ICL compared to the SI–N region across all groups (F: *p* < 0.001; CTRL: *p* < 0.001; OA: *p* < 0.001). Also, larger CD14^+^ areas were found in the SI–V compared to the SI–N region in the CTRL group (*p* < 0.030) and in the ICL compared to the SI–V region in the OA group (*p* < 0.001).

**Fig. 3:**
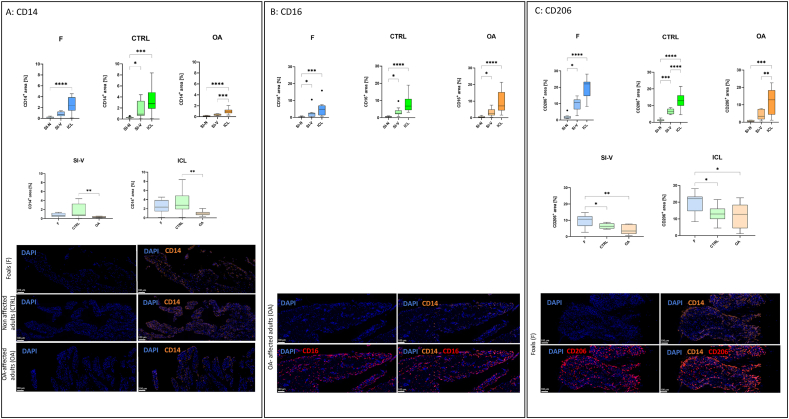
(A)-C)) Immunofluorescence of CD14, CD16, and CD206. Boxplots of the CD14^+^**A)**, CD16^+^**B)**, and CD206+ **C)** area within three specific tissue regions: the SI-N, the SI-V, and the ICL. Fluorescence signals are given as ratio of signal-positive area within each tissue region (in %) for the F group (foals), the CTRL group (control; non-affected adults), and the OA group (OA-affected adults). Significant differences are indicated (∗*p* < 0.050; ∗∗*p* < 0.010; ∗∗∗*p* < 0.001; ∗∗∗∗*p* < 0.0001). For the CD16^+^ area **B)**, there were no significant differences between the three groups (data not shown). Boxplots are supplemented by representative immunofluorescence images of synovial tissues samples within the SI–V region. Applied antibodies are conjugated with AlexaFluor647 (anti-equine CD14), with AlexaFluor555 (anti-equine CD16), and PE (anti-human CD206-PE). DAPI was used as blue-fluorescent DNA stain, indicating cell nuclei. Fluorescence images for CD14^+^**A)** include DAPI staining (left row) and overlay staining of DAPI and CD14 (right row) from all three groups (foal, control, OA-affected adults). Fluorescence images for CD16^+^**B)** include DAPI staining and overlay staining of DAPI and CD14, DAPI and CD16, as well as DAPI with CD14 and CD16 from an OA-affected adult horse (OA). Fluorescence images for CD206+ **C)** include DAPI staining and overlay staining of DAPI and CD14, DAPI and 206, as well as DAPI with CD14 and CD206 from a foal (F).

Group-specific CD14 expression: The CTRL group showed significantly higher CD14^+^ areas compared to the OA group in both the SI–V (*p* < 0.007) and ICL region (p < 0.009) (Fig. 3A).

Tissue region-dependent CD16 and CD206 distribution: Areas of CD16^+^ and CD206+ were consistently elevated in the SI–V and notably pronounced within the ICL when compared to the SI–N across all three groups.

Group-specific CD16 and CD206 expression: CD16^+^ areas (SI–N, SI–V, ICL) were similar between the F, CTRL, and OA groups (Fig. 3B). The F group exhibited higher CD206+ areas in both the SI–V and ICL compared to the CTRL (SI–V: *p* < 0.040; ICL: *p* < 0.040) and the OA group (SI–V: *p* < 0.001; ICL: *p* < 0.020) (Fig. 3C).

The co-localized fluorescence signals (CD14^+^CD16^+^, CD14^+^CD206+) are given as ratios: Within CD14^+^ areas, these could also be positive for either CD16, or CD206, or none of these two markers. This means CD14^+^ is 100 % and the other option is a % of the total CD14^+^ area. So, if CD14^+^ is 10 and CD14^+^CD16^+^ is 8 it would be 80 %. CD14^+^CD16^+^ areas were increased in the OA group when compared to the F group (*p* < 0.020), while similar trends were observed in the other tissue regions and by comparing the OA and the CTRL group (not statistically significant). In CD14^+^CD206+ cells, the OA group exhibited a notably reduced ratio compared to the CTRL and F group within each region. Foals displayed a significantly increased CD14^+^CD206+ area compared to both the CTRL (SI–V: *p* < 0.009; ICL: *p* < 0.010) and OA group (SI–V: *p* < 0.010; ICL: *p* < 0.030) (Supplementary Figure 2).

### 3.3 Cytokine and chemokine analysis

**Fig. 4:**
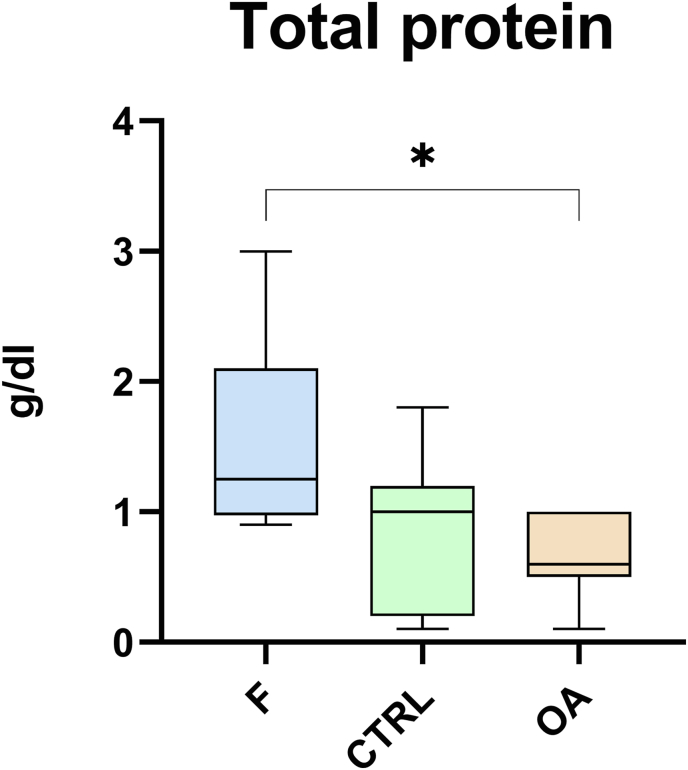
Boxplot of synovial fluid total protein (TP) concentrations. Synovial fluid TP concentration for the F group (foals), the CTRL group (control; non-affected adults), and the OA group (OA-affected adults) are displayed as boxplots. Significant differences are indicated (∗*p* < 0.050).

The cytokines IL-1β, IFN-α, and IL-4 as well as the chemokine CCL3 remained below the limit of detection in all analyzed synovial fluid samples (lower limits of detection: IL-1β: 61 pg/ml; IFN-α: 12 pg/ml; IL-4: 40 pg/ml; CCL3: 5 pg/ml). Synovial fluid concentrations of TNF-α, IFN-γ, IL-17A, IL-10, CCL2, CCL5, and CCL11 showed no significant differences between the three groups (*p* > 0.050) (Table 4). Total protein concentrations were higher in samples of the F group (median 1.3 g/dl, 1–1.95 g/dl) compared to samples of the OA group (median 0.6 g/dl, 0.5–1 g/dl) (*p* < 0.010) (Fig. 4).

**Table 4:** Median (interquartile range) concentrations of cytokines and chemokines in synovial fluid samples from foals (F), non-affected adults serving as control (CTRL), and from OA-affected adults. Statistical differences were not detected (*p* > 0.050). The cytokines IL-1β, IFN-α, and IL-4 as well as the chemokine CCL3 remained below the limit of detection in all analyzed synovial fluid samples (lower limits of detection: IL-1β: 61 pg/ml; IFN-α: 12 pg/ml; IL-4: 40 pg/ml; CCL3: 5 pg/ml).

	Foals (F)	Non-affected adults (control; CTRL)	OA-affected adults (OA)
TNF-α [pg/ml]	2510 (0–19,442.26)	6104 (1566–20,743)	13,979 (2740.5–18,559.5)
IFN-γ [U/ml]	11 (3–14.75)	5 (4–19)	4 (3.5–6)
IL-17A [U/ml]	6.5 (3.5–8.75)	4 (3–6)	4 (3–5)
IL-10 [pg/ml]	103.5 (28.25–161)	58 (34–85)	40 (27–78)
CCL2 [pg/ml]	734.6 (613.25–1598.25)	1282 (352–3061)	992 (701.5–2446)
CCL5 [pg/ml]	236 (107.25–314.5)	90 (37–240)	111 (66–176)
CCL11 [pg/ml]	1636 (741.5–4549.75)	2586 (1561–4007)	3840 (2003.5–6991.5)

## 4 Discussion

Synovial macrophages constitute a complex, heterogeneous, and dynamic cell population, making their in-depth study in horses essential – not only to enhance our understanding of osteoarthritis (OA) pathogenesis [32,33], but also as a therapeutic target, considering the horse as a patient and a translational model for OA [[34], [35], [36]].

This study established immunofluorescence staining of the surface markers CD14, CD16, and CD206 on equine synovial tissue. Notably, CD16 expression on equine synovial tissue has not been previously reported. Marker expression was quantified semi-automatically within defined tissue regions based on histological layers [37,38], enabling distinction of potential tissue macrophages and supporting future research on their origin and migration. Samples included OA-affected and non-OA adult controls, as well as foals, making this the first report to characterize immunofluorescence staining of synovial tissue in foals.

We demonstrated that the joint status of foals and OA-related changes in adult horses alter the CD14^+^ and CD206+ areas. The highest CD14^+^ area was found within the ICL and SI–V, being significantly increased in control horses in both the tissue regions. This contradicts studies by Menarim et al., which showed a significantly higher CD14 expression in OA-affected than in non-affected equine fetlock joints [14]. Although not evaluated in our study, a potentially decreased overall cellularity and progressive fibrosis of the rather chronic OA of the present OA cohort might influence the decreased CD14^+^ areas in OA samples. Furthermore, the CD14-associated fluorescence signal was clearly below 10 % of the area of the respective tissue region and also lower than the CD16^+^ and CD206+ areas, possibly due to inconsistent CD14 staining results or differences in thresholding in the Cy5 (CD14) and Cy3 (CD16 or CD206) channels. Additional validation of these marker combinations will be included in further studies.

While CD14 [[12], [13], [14], [15]] and CD206 [20,21] are established equine macrophage markers, CD16 has not previously been used for equine synovial tissue. Although CD86 and CD80 have been suggested as marker for rather pro-inflammatory macrophages [9,14,20,39,40], immunofluorescence staining of equine synovial tissue exhibited suboptimal signals (anti-CD80 [clone RMMP-1] and anti-CD86 [clone IT2.2, clone 2331 [FUN-1]]; data not shown). In horses, CD16 expression has already been shown on natural killer cells, non-conventional monocytes, and alveolar macrophages [13,[17], [18], [19]]. This low affinity Fc-γ-receptor III (FcγRIII; CD16) can bind IgG immune complexes, which potentially trigger pro-inflammatory pathways during arthritis initiation [41]. Recently, a clear link between CD16^+^ expression and inflammatory cell characteristics of human monocyte subsets has been reported [42]. Like for CD14, the highest CD16^+^ area was detected within the ICL and SI–V. In contrast to the hypothesis that age-related differences may exist in the CD16 expressions in mammals [17], the present study did not confirm this assumption by demonstrating similar CD16^+^ areas. The CD14^+^CD16^+^ area within the ICL was increased in OA-affected samples; notably, statistical significance was only achieved when compared to the F group. Results of these ratios should be interpreted with caution not only because of the limited sample size, but also since CD14^+^ cell areas in both the ILC and SI–V regions were significantly reduced in OA-affected horses. Moreover, CD14 staining might show some variability as reflected by overall small CD14^+^ areas. Therefore, ratios of the areas of signals near the threshold in different channels might be not ideal for analysis of co-localization of different cellular surface markers. These clear limitations of the applied methodological approach make complementary methodological approaches to single cell analyses essential to evaluate the potential of CD16 as equine macrophage marker.

CD206 is a commonly employed macrophage marker [20], whose regulation was demonstrated in equine asthma [13] but not in equine OA [14]. The mannose receptor CD206 involved in scavenging responses is associated with regulatory or anti-inflammatory macrophage phenotypes, as shown in post-traumatic OA-affected murine joints, where CD206+ macrophage populations were increased in a mouse strain resistant to post-traumatic OA [43]. In the present study, CD206+ areas and CD14^+^CD206+ areas in the ICL and SI–V were increased in foals’ synovial tissues compared to CTRL and OA groups. This suggests a role for CD206+ synovial cells in development and maturation of the synovial environment. However, the same methodological limitations as for CD14^+^CD16^+^ areas make future studies essential to precisely conclude the positively stained cells as potential synovial tissue macrophages.

Like the OA group, the F group also revealed a significantly increased intimal lining cell density. In OA-affected synovium, this goes along with an increased cellular infiltration due to inflammation, a finding that is reflected by the significantly increased cellular infiltration in OA samples compared to CTRL samples. In foals, the increased intimal lining cell density might rather be related to maturation, which is a dynamic process involving not only cell proliferation, but also morphological and immunological adaptation of the synovial tissue to required stresses. This contrasts with the findings of della Tommasa et al., who reported lower cell densities in juvenile horses, possibly due to the higher median age of foals included in the present study (median: 39 days; median della Tommasa et al.: 5 days [24]) and different methodology.

Synovial tissues samples were further examined regarding vascularization, reflecting early tissue remodeling during phases of maturation and degeneration. The SI–V revealed an increased number of blood vessels per square millimeter across all groups. While microscopic OARSI scoring showed no significant difference in vascularization between the CTRL and OA groups, the OA group revealed a significantly increased number of blood vessels in the SI–N. This may reflect the fact that, in general, the ingrowth of blood vessels originates from peripheral joint-associated structures and is therefore only visible within OA-affected joints in the periphery SI–N. A previous study also observed an increased number of blood vessels in juvenile horses [24]. Reasons for these different findings, e. g. the older age of foals included in our study, can only be suspected.

The second hypothesis examined whether synovial fluid cytokine and chemokine concentrations match the macroscopic and microscopic joint conditions. Although an average macroscopic OARSI score of 5 indicated moderate osteoarthritic changes in the OA-targeted joints, microscopic scoring of the synovial membrane revealed significant differences only in cellular infiltration when comparing non-affected and OA-affected joints. These results are similar to those of Menarim et al., in which only one parameter (subintimal edema) was found to differ significantly at a microscopic level in fetlock joints with a macroscopic OARSI score of at least 2 per category (moderate OA), and in which lameness was clinically recorded as originating in the fetlock joint [14]. This discrepancy between macroscopic articular cartilage assessment and the microscopic score of synovial tissue underscores the presumably subclinical low-grade inflammatory and potentially intermitted character of synovial inflammation in naturally occurring equine OA in the presented cohort classified by OARSI scoring. Measured synovial fluid cytokine and chemokine concentrations did not correlate with either disease stage or age of the horses, thereby not supporting our second hypothesis. Overall, the detected wide ranges of mediator concentrations are of unknown origin. These interindividual differences might be related to storage effects like reported for human serum mediators [44] or a potential diurnal secretion rhythm [45]. Previous studies including bead-based multiplex assays of synovial fluid revealed increased TNF-α concentrations in both naturally occurring and IL-1β-induced OA compared to non-affected joints. Further, measured chemokine concentrations varied depending on OA-model and revealed temporary responses [29,30]. The limited number of studies with similar detection methods for cytokine and chemokine concentrations in comparable joint pathologies makes it difficult to compare the concentrations both in absolute and relative terms. In summary, it can be concluded that, in the present study cohort in which the presence of fetlock OA was assessed post-mortem using the OARSI scoring, there is no classic synovitis with elevated pro-inflammatory synovial fluid markers given that there were no differences compared with the control group.

Synovial fluid analysis revealed significantly increased TP concentrations in foals when compared to synovia from OA-affected joints. Blood contamination was not visible and excessive dilution with the anticoagulant K2EDTA as the cause of an increased TP concentration can be ruled out (2 ml of synovial fluid was collected in 2 ml sample tubes containing K3EDTA) [46]. Since six of the 10 foals showed signs of septicemia (Supplementary Table 1) and the TP concentrations (median 1.25 g/dl, 1–1.95 g/dl) were similar to those reported for septic foals by Ribera et al. (median 1.80 g/dl, 0.9–2.2 g/dl) [47], increased TP concentrations may be due to septic conditions in the context of a rather permeable and immature blood-synovia barrier, as found in other organs such as the gastrointestinal tract of developing foals [48]. It is not possible to determine conclusively whether the foal's systemic inflammatory response in cases of suspected septicemia has also affected the evaluated parameters or the resident or migrated synovial immune cells. Since septicemia represents a very common condition in foals due to failure of passive transfer and survival rates vary between 26 and 86 % [49], the exclusion of potentially septic foals from post-mortem synovial tissue collection was not possible in the present foal's cohort.

Limitations of the study include the unknown clinical history of the sampled horses, and the categorization based solely on macroscopic joint evaluation. It cannot be determined whether the horses were clinically affected by joint pain. To date, no evidence comparable to that found in other species has emerged to suggest that systemic conditions trigger or modulate equine OA. However, the presence of systemic diseases in the control group potentially influencing joint homeostasis cannot completely be ruled out (Supplementary Table 1). Furthermore, the sample size was limited, as specimens were recruited from three distinct subpopulations with seasonal availability, particularly in the case of foals.

To conclude, the present study expanded the existing immunohistological characterization profile of equine synovial tissue by incorporating a new marker (CD16) and a semi-automated quantitative analysis of immunofluorescence images. Moreover, tissue samples from foals were analyzed for the first time. Based on our findings in the here presented cohort, we propose that equine OA of the metacarpophalangeal joint classified by OARSI scoring is a rather low-grade chronic inflammatory condition with only minor inflammatory histological signs within the synovium and no uniformly increased concentrations of acute inflammatory mediators in the synovial fluid based on cytokine, chemokine, and TP concentrations.

## Author contributions

AT and SPR conceptualized the study and acquired funding. GL and TJT performed the experiments. BW, CLS, and RU analyzed samples and supported method establishment and optimization. KW conceptualized and analyzed data. GL, KW, SPR and AT constructed the manuscript. WB, BW, CLS, SPR and AT edited the manuscript. All authors contributed to the article and approved the submitted version.

## Role of the funding source

This work was funded by the Junior Scientist Support Program financed by the Freundeskreis Tiermedizin of the Faculty of Veterinary Medicine of Leipzig University, and by Ceva Santé Animale (grant recipient: SPR). Furthermore, this work was supported by the Open Access Publishing Fund of Leipzig University.

## Conflict of interest

The authors declare that the research was conducted in the absence of any commercial or financial relationships that could be construed as a potential conflict of interest.
