# Supplementary material for: Immunohistological characterization of equine synovial tissue in metacarpophalangeal joints of different ages and osteoarthritis status
Source: Osteoarthr Cartil Open. 2026 May 14;8(3):100819. doi: 10.1016/j.ocarto.2026.100819 (PMC13213949; doi:10.1016/j.ocarto.2026.100819)
Supplement: Multimedia component 1 [file mmc1.docx]

**Supplementary Figure 1. Macrophage marker validation on equine monocyte-derived macrophages differentiated and stimulated *in* vitro.** Representative immunofluorescence images of equine peripheral blood mononuclear cells (PBMC) isolated via density gradient centrifugation and differentiated in **(A-F)** granulocyte-macrophage colony-stimulating factor (GM-CSF; equine recombinant, Biomol GmbH)- or **(G-L)** macrophage colony-stimulating factor (M-CSF; bovine recombinant, Biomol GmbH)-supplemented medium (Dulbecco’s modified Eagle medium 1 g glucose/L [Gibco™ by Thermo Fisher Scientific, Life Technologies] supplemented with 10 % fetal bovine serum [Gibco™ by Thermo Fisher Scientific, Life Technologies], 1 % penicillin streptomycin [Sigma-Aldrich® by Merck KGaA] and 0.1 % gentamycin [Carl Roth GmbH + Co. KG]) for 6 days including one change of media, followed by classical (IFN-γ [equine recombinant, Biomol GmbH] and LPS [from *Escherichia coli*, Sigma-Aldrich® by Merck KGaA], 100 ng/ml each;) or alternative activation (IL-4 [equine recombinant, Biomol GmbH] and IL-13 [equine recombinant, Biomol GmbH], 200 ng/ml each) for 24 hours. Control samples were not stimulated. Direct immunofluorescence staining was performed to analyze CD14, CD16 and CD206 expression (anti-equine CD14, clone 105, 1:500 dilution; anti-equine CD16, clone 59G5, 1:100 dilution; anti-human CD206-PE, clone 3.29B1.1, 1:40 dilution). CD14 was conjugated in house with Alexa 657 (displayed yellow), and served as pan macrophage marker, DAPI (displayed blue) was used as nuclear counterstain. CD16 was conjugated in house with Alexa 555 and CD206 was pre-conjugated with PE (both displayed red). **C** and **F** display an overlay of GM-CSF-differentiated and classically stimulated PBMC; **I** and **L** show an overlay of M-CSF-differentiated and alternatively stimulated PBMC. **M** For each stained sample, 100 cells were differentiated, and the percentages of CD16+ and CD206+ cells were evaluated. Fluorescence signals that completely surrounded the respective cell nucleus were rated as ‘high’, while those that only partially surrounded it were classified as ‘low’. GM-CSF differentiation with classical activation (pro-inflammatory, M1-like) induced CD16 increase while M-CSF differentiation and alternative activation (anti-inflammatory, M2-like) induced CD206 expression.

| **GM-CSF differentiation + stimulation** **IFN-γ 100 ng/ml, LPS 100 ng/ml** | | |
| --- | --- | --- |
| **A**  **B**  **C**  CD14  DAPI  CD16  DAPI  CD14  CD16  DAPI 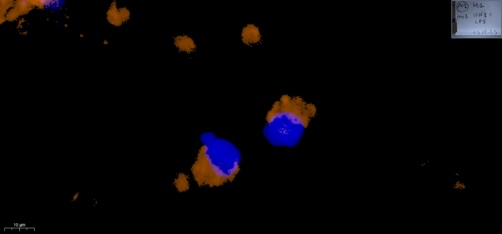 10 µm | 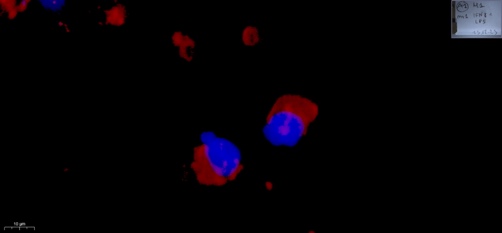 10 µm | 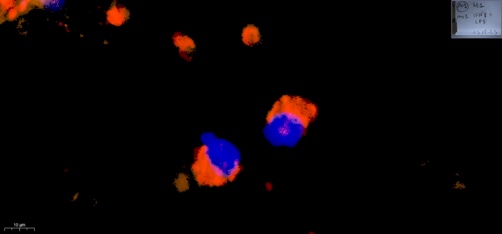 10 µm |
|  |  |  |
| **D**  **E**  **F**  CD14 DAPI  CD206 DAPI  CD14 CD206 DAPI 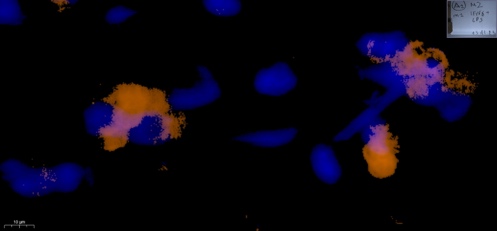 10 µm | 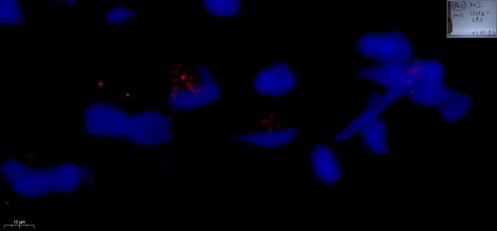 10 µm | 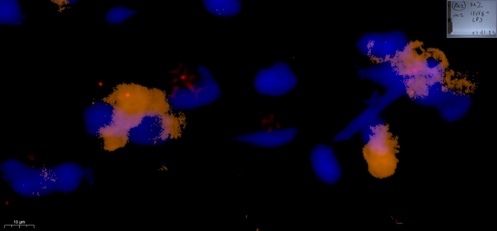 10 µm |
|  |  |  |
| **M-CSF differentiation + stimulation IL-4 200 ng/ml, IL-13 200 ng/ml** | | |
| **H**  **I**  **G**  CD14  DAPI  CD16  DAPI  CD14  CD16  DAPI 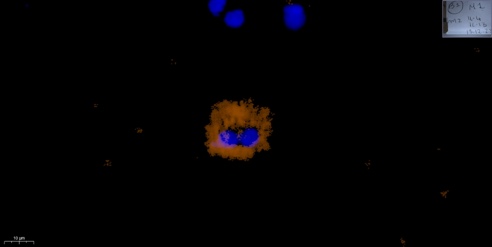 10 µm | 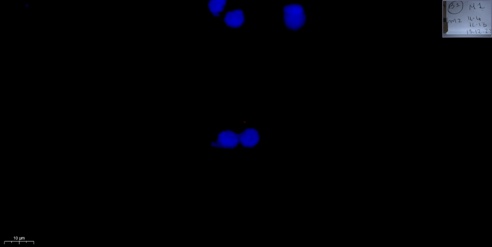 10 µm | 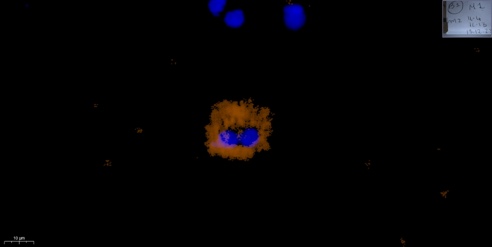 10 µm |
|  |  |  |
| **K**  **J**  **L**  CD14 DAPI  CD206 DAPI  CD14  CD206  DAPI 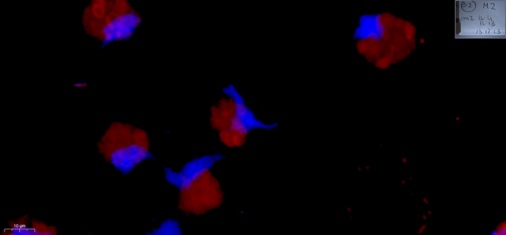 10 µm | 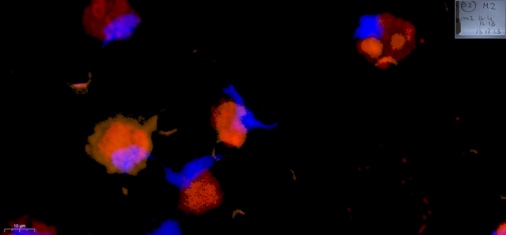 10 µm | 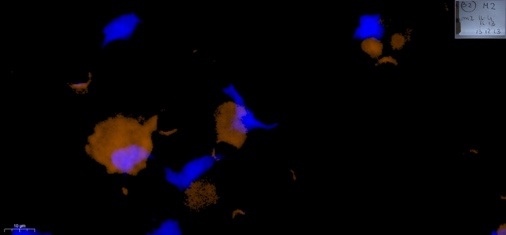 10 µm |
|  |  |  |

| **M** |  |  |  |  |  |  |
| --- | --- | --- | --- | --- | --- | --- |
| Differentiation / Stimulation |  |  |  |  |  |  |
| GM-CSF | CD16^+high^ | CD16^+low^ | CD16^-^ | CD206^+high^ | CD206^+low^ | CD206^-^ |
| IFN-γ, LPS | **70 %** | 13 % | 17 % | 19 % | 16 % | **65 %** |
| w/o | **51 %** | 31 % | 18 % | 38% | 20% | **42 %** |
|  |  |  |  |  |  |  |
| M-CSF | CD16^+high^ | CD16^+low^ | CD16^-^ | CD206^+high^ | CD206^+low^ | CD206^-^ |
| IL-4, IL13 | 18 % | 22% | **60%** | **85 %** | 10 % | 5 % |
| w/o | 17 % | 28 % | **55 %** | **48 %** | 20 % | 32 % |
