# Supplementary material for: Immunohistological characterization of equine synovial tissue in metacarpophalangeal joints of different ages and osteoarthritis status
Source: Osteoarthr Cartil Open. 2026 May 14;8(3):100819. doi: 10.1016/j.ocarto.2026.100819 (PMC13213949; doi:10.1016/j.ocarto.2026.100819)

**Supplementary Figure 2. Rations of CD14+CD16+ and CD14+CD206+ areas within CD14+ areas of synovial macrophages distinguish foals’ and osteoarthritic joints.** Within three joint tissue regions, the subintimal layer not associated with the villous region (SI-N), the subintimal tissue layer of the villous region (SI-V), and the intimal cell layer (ICL), fluorescence signals are given as ratio of CD14+CD16+ (**A**) or CD14+CD206+ (**B**) positive areas within each CD14 signal-positive area (in %) for the foals group (F), the non-affected adult group (CTRL), and the adult, osteoarthritis-affected group (OA). Significant differences are indicated (* *p* < 0.050; ** *p* < 0.010).

**A: CD14, CD16**

**B: CD14, CD206**


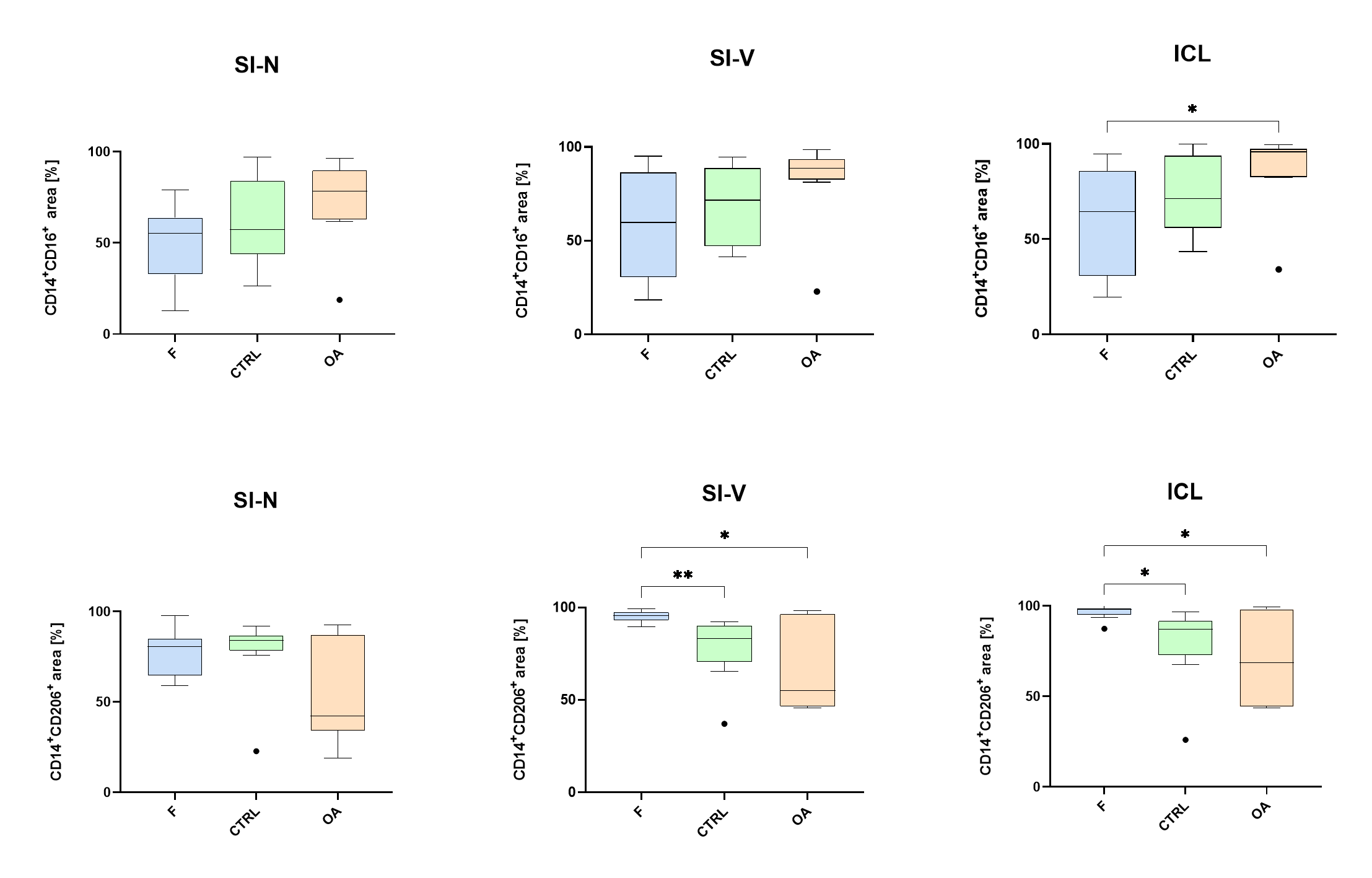

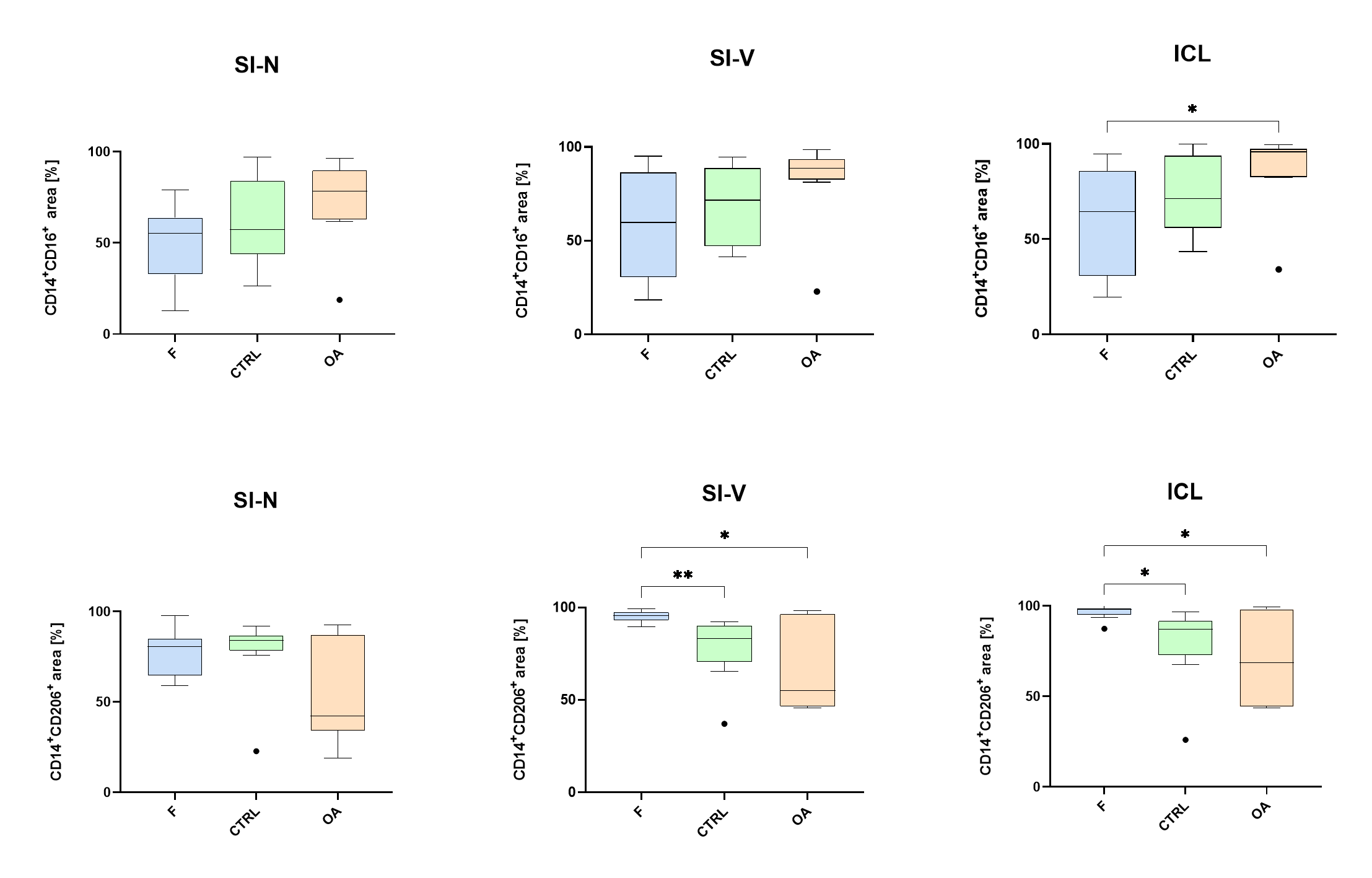

Supplement: Multimedia component 2 [file mmc2.docx]
