# Supplementary material for: Immunohistological characterization of equine synovial tissue in metacarpophalangeal joints of different ages and osteoarthritis status
Source: Osteoarthr Cartil Open. 2026 May 14;8(3):100819. doi: 10.1016/j.ocarto.2026.100819 (PMC13213949; doi:10.1016/j.ocarto.2026.100819)
Supplement: Multimedia component 3 [file mmc3.docx]

**Supplementary Table 1.** Detailed overview (age, sex, reason for euthanasia/death, potential for septicemia) of included foals (F) as well as detailed overview (age, sex, reason for euthanasia, intended use) of included control (CTRL) and OA-affected (OA) adult horses.

| **Foals (F group)** *n* = 10 | | | | |
| --- | --- | --- | --- | --- |
| **Sample number** | **Age**  **[days]** | **Sex** | **Reason for euthanasia/death** | **Septic** |
| **1** | 1 | male | - dystocia due to maldisposition   (no sign of pre- and dysmaturity or of being ‘post-term’; no vital signs at presentation) | no |
| **2** | 1 | female | - dystocia due to maldisposition   (no sign of pre- and dysmaturity or of being ‘post-term’; no vital signs at presentation) | no |
| **3** | 78 | female | - trauma right tarsal region with septic arthritis of the talocrural joint   (presentation: 10 days after traumatic insult; euthanasia: 18 days after presentation) | yes |
| **4** | 7 | female | - trauma medial coronary band left front limb with septic arthritis of the proximal interphalangeal joint and rupture of the medial collateral ligament   (presentation: 1 day after suspected traumatic insult; euthanasia: on presentation | yes |
| **5** | 48 | male | - closed olecranon fracture typ 1a (presentation: 14 days after traumatic insult; euthanasia: 1 day after presentation) | no |
| **6** | 30 | male | - omphalitis with persistent urachus - septic arthritis of the left femoropatellar joint, lateral and medial femorotibial joints   (presentation: 3 days after onset of clinical symptoms of lameness; euthanasia: 22 days after presentation) | yes |
| **7** | 17 | female | - septic shock   (presentation: 2 days after onset of clinical symptoms of patent urachus; death: peracute death 8 hours after presentation) | yes |
| **8** | 72 | female | - closed proximal physeal fracture Salter Harris type II with osteomyelitis following surgical site infection (internal fixation)   (presentation: same day as observed trauma; euthanasia: 15 days after presentation) | yes |
| **9** | 70 | female | - cellulitis, osteitis and osteomyelitis with sequestration of the right hind third metacarpal bone after surgical treatment of an open, infected midbody fracture of the fourth metacarpal bone   (presentation: 13 days after observed trauma; euthanasia: 14 days after presentation) | yes |
| **10** | 126 | male | - strangulating lesion of the small intestine   (presentation: 1 day after onset of clinical symptoms of colic; euthanasia: at presentation) | no |
| **Adult control horses (CTRL)** *n* = 11 | | | | |
| **Sample number** | **Age**  **[years]** | **Sex** | **Reason for euthanasia** | **Intended use** |
| **1** | 13 | female | - acute trauma right front limb involving the digital flexor tendon sheath, the superficial digital flexor tendon and the third metacarpal bone   (euthanasia at presentation; no pre-existing conditions, in care of the owner since foalhood) | leisure riding |
| **2** | 9 | female | - acute colic (large colon displacement with tympany; conservative treatment)   (euthanasia 2 days after presentation; no pre-existing conditions, owned since birth) | show jumping |
| **3** | 5 | gelding | - 2 weeks prior to presentation signs of severe ataxia grade 4-5/5 (Mayhew ataxia scale) after returning from pasture (infectious diseases tested negatively, no pyrexia), arthropathy facet joints C5 to T1, suspected spinal stenosis C6-C7, suspected fracture facet joint C7-T1)   (euthanasia 6 days after presentation; no pre-existing conditions, owned since age of 3 years) | leisure riding |
| **4** | 13 | male | - acute colic (right sided acquired inguinal hernia; surgical herniorrhaphy with hemicastration)   (euthanasia 6 days after presentation due to post-operative ileus; no pre-existing conditions, owned since birth) | leisure riding |
| **5** | 17 | female | - ovariectomy due to left sided granulosa cell tumor with acute hemoperitoneum   (euthanasia 18 days after presentation due to laminitis and surgical site infection; chronic cellulitis right hind limb since age of 11 years, PPID since age of 16 years, owned since age of 8 years) | leisure riding |
| **6** | 1 | female | - acute trauma of the right stifle region with open transverse fracture of the right patella and suspected involvement of the femorotibial joints   (euthanasia at presentation) | not yet in training |
| **7** | 5 | female | - acute colic (epiploic foramen entrapment and partial small intestinal volvulus; surgical replacement without resection)   (death during recovery due to cardiac arrest; no pre-existing conditions) | show jumping |
| **8** | 19 | gelding | - lymphoma (symptoms 4 weeks prior to presentation)   (euthanasia 1 day after presentation; no pre-existing conditions, owned since birth) | leisure riding |
| **9** | 9 | female | - 3-days-old trauma right hind limb with septic calcaneal bursitis and septic tendinitis (surgical bursoscopy)   (euthanasia 10 days after presentation; no pre-existing conditions, owned since age of 6 years) | leisure riding |
| **10** | 1 | male | - 6-days-old puncture wound left front limb involving deep digital flexor tendon, navicular bursa and navicular bone   (euthanasia 1 day after presentation; no pre-existing conditions) | not yet in training |
| **11** | 10 | female | - acute trauma of the left front hoof involving the hoof capsula, distal phalanx, ungular cartilage and deep digital flexor tendon   (euthanasia at presentation; no pre-existing conditions, owned since birth) | brood mare |
| **Adult OA-affected horses (OA)** *n* = 9 | | | | |
| **Sample number** | **Age**  **[years]** | **Sex** | **Reason for euthanasia** | **Intended use** |
| **1** | 24 | gelding | - acute rectal tear grade IV with peritonitis   (euthanasia at presentation; no pre-existing conditions) | leisure riding |
| **2** | 19 | female | - 2 weeks prior to presentation signs of moderate ataxia grade 3/5 (Mayhew ataxia scale), osteoarthritis intervertebral joint C6-C7 with suspected fracture of C7, suspected spinal stenosis C6-C7, arthropathy facet joints C6-C7   (euthanasia 1 day after presentation; osteoarthritis of the tarsometatarsal- and distal intertarsal joints) | leisure riding |
| **3** | 24 | gelding | - 3-weeks-old dysphagia, trauma with tearing of the soft palate, secondary sinusitis   (euthanasia 2 days after presentation; owned since age of 12 years) | leisure riding |
| **4** | 8 | gelding | - acute colic (right dorsal displacement of the large colon; surgical replacement with enterotomy)   (euthanasia 10 days after presentation due to post-operative ileus, rupture of the esophagus and aspiration pneumonia; owned since age of 7 years) | leisure riding |
| **5** | 16 | female | - 3-days-old puncture wound right hind limb involving deep digital flexor tendon   (euthanasia 26 days after presentation due to uncontrolled infection of the deep digital flexor tendon; no pre-existing conditions, owned since age of 14) | leisure riding |
| **6** | 16 | female | - osteoarthritis left front metacarpophalangeal joint (subchondral cystic lesion proximomedial first phalanx, bone-edema like-lesion of the first phalanx)   (euthanasia 3 days after presentation due to severe non-responding lameness; no pre-existing conditions, owned since age of 3 years) | leisure riding |
| **7** | 26 | gelding | - acute colic (strangulating obstruction of the small intestine)   (euthanasia at presentation; no pre-existing conditions, owned since age of 4) | leisure riding |
| **8** | 17 | female | - acute colic (strangulating obstruction of the small intestine)   (euthanasia at presentation; iliosacral dysfunction) | leisure riding |
| **9** | 13 | gelding | - acute colic (non-strangulating obstruction of the small intestine; conservative treatment)   (euthanasia 2 days after presentation due to ongoing symptoms; no pre-existing conditions, owned since birth) | leisure riding |
